# The Role of Background Acoustic Stimuli in Dual Tasks: A Study on Postural Control Performance

**DOI:** 10.1002/brb3.70750

**Published:** 2025-08-12

**Authors:** Selin Sarıçamlık, Nizamettin Burak Avcı, Öznur Yiğit

**Affiliations:** ^1^ Audiology, Faculty of Health Sciences Atilim University Ankara Türkiye; ^2^ Audiology, Faculty of Health Sciences Trakya University Edirne Türkiye; ^3^ Audiology, Faculty of Health Sciences Hacettepe University Ankara Türkiye

**Keywords:** multitasking, posture, selective attention, Stroop task

## Abstract

**Introduction:**

Performing everyday tasks requires the use of multiple cognitive, sensory, and emotional systems. The interference of different variables in these multitasking systems affects our motor‐balance system. This study was conducted to investigate how acoustic stimuli presented during a cognitive‐motor dual task affect postural control in healthy young adults.

**Methods:**

Fifty‐four healthy participants (39 females, 15 males; total age 21.87±1.18, range 19–24) were randomly assigned to control (silent), noise (multi‐talker babble), or music (Mozart‐Jupiter) groups based on testing environment. During the Stroop test, conducted with acoustic stimuli, postural sway velocity was measured on firm and foam surfaces with eyes open. The dual‐task effect was assessed using the Wilcoxon test, and group comparisons employed one‐way ANOVA or Kruskal‐Wallis tests. Independent t tests and Mann‐Whitney U tests were used for two‐group comparisons. Statistical significance was set at p<0.05 (Bonferroni‐adjusted p<0.017).

**Results:**

The silent cognitive‐motor dual task increased postural sway on firm (median increased from 0.18 to 0.26 deg/s) and foam (median increased from 0.21 to 0.32 deg/s) surfaces. Music did not significantly affect cognitive performance or postural sway compared to the control group. However, noise reduced postural sway on firm and foam surfaces compared to the control group but did not affect cognitive performance. There was no significant difference in average Stroop response times between the groups or between the firm and foam surface comparisons.

**Conclusions:**

During inhibitory control tasks, cognitive effort prioritized in young people in easy‐to‐balance situations. Background noise affects motor‐cognitive interaction, highlighting its potential for enhancing vestibular rehabilitation strategies in multitasking and guiding future research.

## Graphical abstract

During the inhibition control task, the group exposed to background noise demonstrated better postural control than the groups exposed to music or silence. Music had no significant impact on cognitive or postural performance. These results emphasize the different effects that various acoustic stimuli have on balance when cognitive load is present.

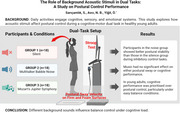

## 1 Introduction

The balance system employs visual, somatosensory, and vestibular information to keep the body's center of pressure and center of gravity constant in proportion to its initial position. While balance is typically automatic and reflexive, it requires conscious effort in challenging situations (Huang et al. 2009). The relationship between attentional focus and balance has been explored through the “Constrained Action Hypothesis,” which suggests that conscious intervention in movement and balance can suppress the motor system's self‐organization, leading to increased postural sway (Wulf et al. 2001).

To better understand cognitive involvement in balance control, researchers have employed dual‐task paradigms, where individuals simultaneously perform a primary motor task and a secondary cognitive task. The cognitive distribution of simultaneously performed tasks has been explained by various theories. The “Capacity Limit Theory” argues that cognitive resources are used by dividing cognitive resources between tasks (Tombu and Jolicoeur 2005), while “Central Bottleneck Theory” suggests that task prioritization dictates resource distribution (Strobach and Torsten 2017). The “Cross‐talk Model” argues that how much attention two tasks need depends on whether they use the same or different parts of the brain, with more confusion happening when different parts are used (Navon and Miller 1987).

Balance‐cognition dual‐task assessments can focus on the second task's changes or both tasks' performance. Kinematic features of walking, gait initiation, and postural control assessments are frequently used as primary motor tasks. Typically, the impact of the cognitive task on postural control is evaluated by analyzing postural sway velocity and center‐of‐gravity (Bayot et al. 2018). The “Nonlinear Interaction Model” describes a U‐shaped relationship between cognitive load and postural performance. Postural sway decreases during easier cognitive tasks. The increase in attentional demand caused by more difficult cognitive tasks increases cognitive effort and negatively affects postural control (Lacour et al. 2008). More recently, the “Synergy Model” proposed by Bonnet and Baudry (2016) suggests that during dual‐task conditions requiring conscious sensory processing, the central nervous system integrates sensory and postural control as a synergistic system rather than independently managing each component.

While most research on cognitive‐motor interactions has focused on visual and somatosensory inputs, emerging evidence suggests that auditory cues also influence postural control and cognitive processing (Shayman et al. 2020). Although acoustic stimuli have been shown to enhance both static (Xu et al. 2018) and dynamic (Shayman et al. 2020) balance, high‐frequency and high‐intensity exposures may negatively affect postural control (Ylikoski et al. 1988). It has been observed that acoustic stimuli with highly repetitive rhythmic information elicit involuntary movements in the body (Senn and Kilchenmann 2015), whereas music with a calmer and more dynamic structure reduces postural sway (Waer et al. 2023). The cognitive effects of auditory stimuli are influenced by the emotional and psychological responses they evoke (Husain et al. 2002). While white noise has been shown to enhance both postural stability (Bartczyk et al. 2019) and cognitive performance (Angwin et al. 2017), multi‐talker noise and complex auditory environments (e.g., environmental or speech noise) may impair both functions (Carr et al. 2020).

Despite the growing interest in cognitive‐motor interactions, the role of auditory stimuli in multitasking remains underexplored. Previous studies have reported conflicting findings regarding the effects of cognitive load on postural control—some supporting the Nonlinear Interaction Model (Ceyte et al. 2014), while others are more consistent with the Constrained Action Hypothesis (Lajoie, Richer, Jehu, and Tran 2016). Collectively, these contrasting results—spanning various age groups and motor‐cognitive task configurations—highlight the complex interplay between cognitive load and postural control. They further suggest that factors such as task difficulty and attentional demands may influence the dominance of one theoretical model over the other. While the “Constrained Action Hypothesis” and related models (e.g., Capacity Limit Theory, Central Bottleneck Theory, and Synergy Model) have sought to explain cognitive‐motor interaction, the role of acoustic stimuli in everyday multitasking contexts remains relatively underexplored. This study was motivated by the need to clarify how acoustic stimuli interact with cognitive and motor processes during dual‐task performance, particularly in relation to postural control. Specifically, we aimed to examine the impact of acoustic stimuli on balance performance under dual‐task conditions in healthy young adults. To our knowledge, this is among the few studies addressing acoustic interference within a dual‐task framework.

## 2 Materials and Methods

This cross‐sectional study was conducted at Hacettepe University, Faculty of Health Sciences, Department of Audiology, with the approval of the Hacettepe University Non‐Interventional Clinical Research Ethics Committee (GO 22/974) in accordance with the Principles of the Declaration of Helsinki.

### 2.1 Participants

As a result of the power analysis, the sample size of this study was determined to be a total of 48 people to find a significant effect size of 0.4714045 units with a test power of 80% and a first‐type error of 5%. The effect size used in the power analysis (*f* = 0.4714045, ∼0.5) is based on an *η* ^2^ value of 0.1, which represents a medium effect and aligns with Cohen's classification (Cohen 1988). This assumption was adopted in the study design, as previous literature involving similar samples has reported medium‐sized effects (Majewska et al. 2017). However, a total of 54 people were included considering the data extraction situation.

A total of 54 volunteer participants aged 19–24 years, with hearing thresholds within normal limits (125–8000 Hz < 15 dBHL), and without any audiovestibular, cognitive, musculoskeletal, or psychological disorders were included in the study. The exclusion criteria were demonstrating abnormal balance function in at least one condition in the Modified Clinical Test of Sensory Interaction in Balance (mCTSIB), a score of 16 or higher on the Dizziness Handicap Inventory (DHI) (Jacobson and Newman 1990), neuropsychiatric drug use, chronic sleep disorders, and color blindness. In addition, to rule out possible effects on balance and cognitive performance, individuals who consumed no more than 0–7 drinks of alcohol per week (Snetselaar et al. 2021), had no substance abuse (Chiao et al. 2024), had a daily sleep duration of at least 6 h (Durmer and Dinges 2005), and had eaten within 3 h before the test were included. The study included one control group (tested in a silent environment) and two experimental groups (tested in noisy and musical environments, respectively). We randomly assigned participants (*n* = 18 per group) to one of the three experimental groups using a computer‐generated simple randomization sequence.

### 2.2 Posturography

The mCTSIB test, implemented via the NeuroCom Balance Master Version 8.2.0 within the Natus Basic Balance Master Static Posturography system, includes four conditions: firm surface with eyes open, firm surface with eyes closed, foam surface with eyes open, and foam surface with eyes closed. Since the Stroop test is visually driven, it can only be administered with eyes open; therefore, only Conditions 1 and 3 were used during Stroop testing. The system records up to three 10‐s trials for each condition. Outcome measures are based on the sway velocity of the center of gravity, calculated by the system along the *x*‐ and *y*‐axes for each condition.

### 2.3 Stroop Stimuli

In our study, we used the color name‐ink mismatch condition, in which the disturbing effect of the Stroop test was observed to be higher (Scarpina and Tagini 2017). Incongruent Stroop stimuli (Red, Blue, Yellow, and Green) in 17 × 30 size according to the name and ink color on a black background are provided with a computer screen placed 100 cm away from the eye level. Stroop stimuli were presented to all individuals in the same order. Since there was no time restriction between Stroop stimuli in our study, mean response time was used to evaluate the effects of different acoustic stimuli on Stroop performance (Scarpina and Tagini 2017). Stroop stimuli were presented from the beginning of the test, moving to the next stimulus as the participant responded. Mean Stroop response time was determined by calculating the average of manually recorded response times for each Stroop stimulus during the three trials of Conditions 1 and 3. The total response time for each condition was divided by the number of valid responses to obtain the mean. Although both correct and incorrect responses were included in the calculation of response time, accuracy rates were analyzed separately and used only to verify participants’ sustained attention during the task. All participants demonstrated over 90% accuracy in each condition of the Stroop task, further supporting the validity of the response time data.

### 2.3 Acoustic Stimuli

Mozart's Symphony No. 41 in C Major “Jupiter” was chosen as the music stimulus in our study due to its positive effects on both balance (Waer et al. 2023) and cognitive performance (Kim 2022). To maintain a comfortable listening level, the part of the Jupiter composition with more stable spectral characteristics between the 14th and 26th min interval was selected. Multi‐talker Babble noise was chosen for its distracting effect and to create a realistic simulation (Gökçen Kesici 2022). In this study multi‐talker babble noise consisting of Turkish words spoken by three different male and three different female speakers at a 7 dB signal‐to‐noise ratio (SNR) was used to observe the distractor effect. Since both acoustic stimuli used in the study contain spectral characteristics that change over time, in order not to move away from the participants' comfortable listening level, the stimuli were presented to the participants with a sound level of 70 dBSPL at maximum amplitude (with Sennheiser HD380 pro headphones through a Lenovo Ideapad laptop). Sound levels from the headphones were measured and adjusted with a sound level meter. The study group continuously presented acoustic stimuli to the participants from the start to the end of the test.

### 2.4 Procedure

All volunteer participants signed the informed consent form. To be evaluated for inclusion criteria, participants were first asked to complete the Demographic Information Form, then pure tone audiometric evaluation was performed with the Maico MA42 audiometer to confirm that hearing thresholds were normal (125–8000 Hz air conduction hearing thresholds of 15 dB or better each, 500–4000 Hz bone conduction hearing thresholds of 15 dB or better each). The four conditions of the mCTSIB test and the DHI questionnaire were completed to assess balance function and perception of disability due to dizziness. Participants who met the inclusion criteria were tested 15 days later to avoid a learning effect in Conditions 1 and 3 of the mCTSIB test with a simultaneous Stroop test. Participants completed this phase in a silent, musical, or noisy environment, depending on their assigned group.

During the mCTSIB test, participants were instructed to look straight ahead with their eyes and body in the same direction, stand in an upright posture with hands at their sides, and say the colors of words presented in the field of view. In order not to restrict the participants’ sway, they were instructed only to keep their feet positioned and to stand upright with their hands at their sides. Before the test, all groups, including the control group, were given headphones that were not removed. Immediately after positioning their feet on the relevant part of the platform, the participants completed a Stroop familiarization session consisting of 10 words. Once participants were sure that they understood the task, three 10‐s recordings were made for each condition of the mCTSIB test in the eyes‐open firm surface (Condition 1) and eyes‐open foam surface (Condition 3).

### 2.5 Statistical Analysis

Statistical analyses were performed with SPSS Statistics v23.0 program. As a result of checking for normal distribution using tests and histograms, all variables showed a normal distribution except for the dual‐task firm surface and dual‐task foam surface variables. Descriptive statistics for normally distributed variables are reported as mean ± standard deviation, while non‐normally distributed variables are presented as median and interquartile range (IQR). For comparisons between two independent groups, the independent samples t‐test was used for normally distributed data, and the Mann–Whitney *U* test was used for non‐normally distributed numerical data. In comparisons between two dependent groups, the Wilcoxon signed‐rank test was applied for non‐normally distributed data. For comparisons among more than two independent groups, one‐way analysis of variance (ANOVA) was used for normally distributed numerical data, and the Kruskal–Wallis test was employed for non‐normally distributed data. In post hoc analysis, pairwise comparisons were conducted using the Mann–Whitney *U* test. A *p* value of < 0.05 was considered statistically significant. For post hoc comparisons, Bonferroni correction was applied, and a *p* value of < 0.017 was considered significant.

## 3 Results

The group that completed the study in a silent environment was named “Control,” the study groups were named “Noise” and “Music.” Age and gender match the groups (Table 1). In addition, mCTSIB results obtained during the baseline (silent and no‐task) balance assessment were compared across the three groups, and no statistically significant differences were found (*p* > 0.05). This indicates that the groups were comparable in terms of static balance performance prior to the experimental phase.

**TABLE 1:** Demographic information of the study sample (*N* = 54).

Group		Age (year)			Gender		
	*n*	Mean ± SD	Min–max	*p* ^a^	Female *n*	Male *n*	*P* ^b^
Control	18	21.61 ± 1.14	19–24	0.124	13	5	0.758
Noise	18	21.67 ± 1.18	20–23		12	6	
Music	18	22.33 ± 1.13	20–24		14	4	
Total	54	21.87 ± 1.18	19–24		39	15	

Abbreviation: SD, standard deviation.

^a^Anova.

^b^Chi‐square test.

### 3.1 Effects of Different Acoustic Stimuli Presented With Stroop Task on Postural Sway Velocity

**FIGURE 1:**
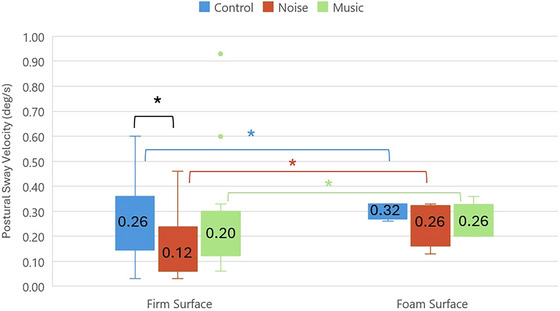
Comparison of postural sway velocity on firm and foam surface with Stroop task. *p < 0.05: Statistically significant.

A statistically significant difference was observed among the three groups in sway velocity under different acoustic conditions presented with the Stroop task on the firm surface (*p* < 0.05). Specifically, in the task condition on the firm surface, the control group exhibited significantly higher sway velocity compared to the noise group (*p* = 0.016). However no significant differences were found between the control and music groups or music and noise groups (*p* > 0.017). These results are graphically illustrated in Figure 1.

There was no significant difference between the three groups in the different acoustic stimuli presented during the dual task on the foam surface (*p* > 0.05). Therefore, two group comparisons were not made for the foam surface condition with Stroop task. Table 2 shows the data on the comparison of sway velocities between the three groups. In all groups, the mean sway velocity showed a significant difference between the firm surface and foam surface conditions (*p* < 0.05).

**TABLE 2:** Stroop tasked postural sway velocity in firm and foam surface conditions.

	Postural Sway Velocity (deg/s)				
Group	Firm surface		Foam surface		*p* ^a^
	Median	75%–25%	Median	75%–25%	
Control	0.26	0.36–0.16	0.32	0.33–0.26	**0.005** ^*^
Noise	0.12	0.23–0.06	0.26	0.33–0.23	**0.002** ^*^
Music	0.20	0.30–0.13	0.26	0.30–0.20	**0.046** ^*^
p^b^	**0.048** ^*^		0.087		

^a^Wilcoxon Test.

^b^Kruskal Wallis Test.

*p < 0.05: statistically significant.

### 3.2 The Effect of the Dual Task on the Postural Sway Velocity

**FIGURE 2:**
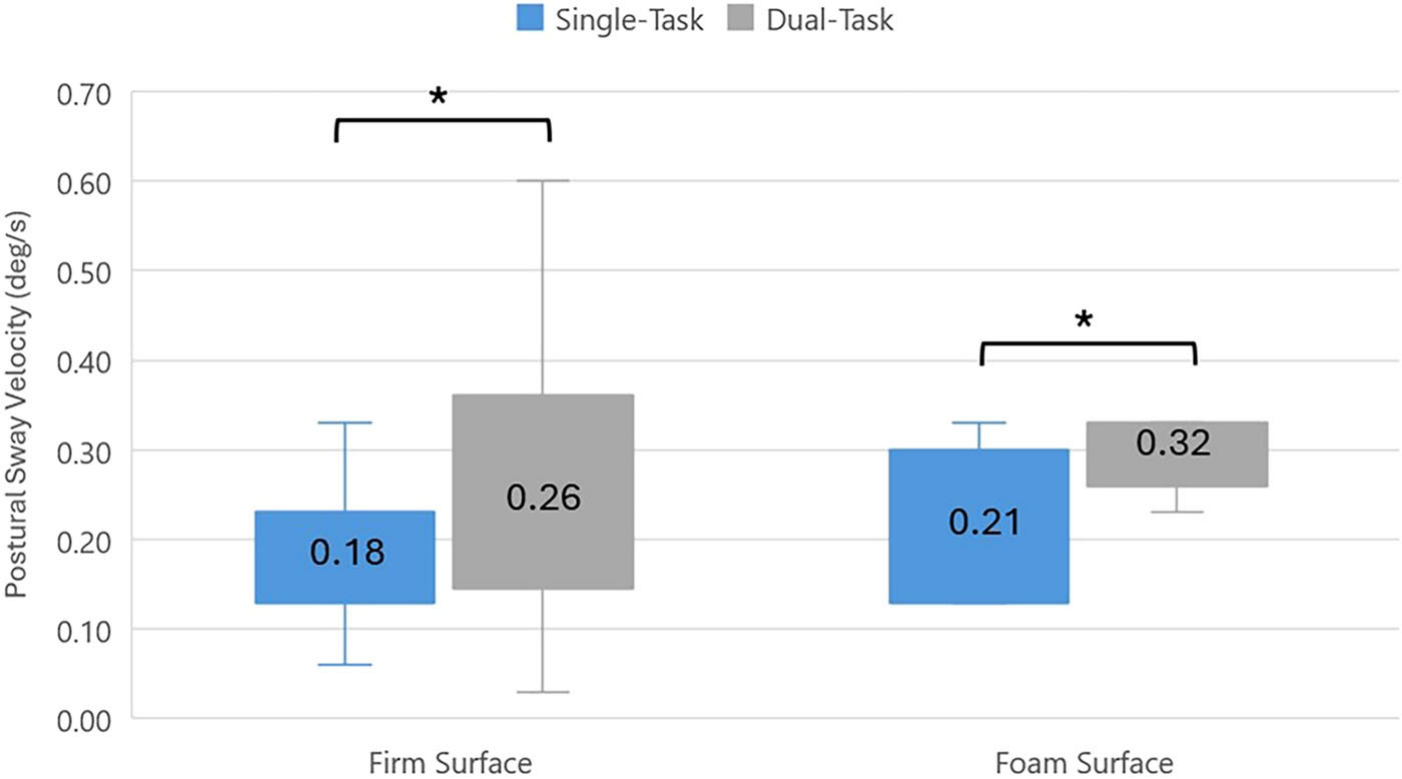
The effect of dual task on postural sway in silent environment (control group). *p < 0,05: Statistically significant.

The effect of the Stroop task on postural sway was examined by comparing the sway velocities of 18 participants in the control group under single‐task and dual‐task conditions in a silent environment. The Stroop task significantly increased postural sway both on the firm surface (*p* < 0.047) and on the foam surface (*p* < 0.001) (Figure 2).

### 3.3 Effects of Acoustic Stimuli on Stroop Mean Response Time

Stroop response time refers to the duration between the presentation of a Stroop stimulus and the participant's corresponding response. No statistically significant differences were observed among the three groups in terms of mean Stroop response times under either condition. (Table 3).

**TABLE 3:** Average Stroop reaction time of the groups in firm surface and foam surface conditions.

	Average Stroop response time (s)	
Group	Firm surface	Foam surface
	Mean ± SD	Mean ± SD
Control	1.31 ± 0.11	1.30 ± 0.12
Noise	1.26 ± 0.19	1.26 ± 0.16
Music	1.28 ± 0.13	1.29 ± 0.14
p	0.629	0.588

*Note: p*: Anova.

Abbreviations: s: second, SD: standard deviation.

## 4 Discussion

In this study, background music presented during the dual task did not significantly affect either cognitive performance or postural control. In contrast, background noise resulted in a significant reduction in postural sway compared to both the music and silent conditions. However, neither music nor noise had a significant impact on mean Stroop response time. The lack of significant group differences in Stroop response times may be attributable to the fixed time window determined by the mCTSIB test duration, which ensured that participants were exposed to a similar number of stimuli. This supports the methodological consistency of the cognitive task across groups.

The present study did not observe any impact of soothing classical music on cognitive or postural outcomes under dual‐task conditions. The therapeutic properties of Mozart's compositions in neurological diseases have been widely discussed in the literature (Dastgheib et al. 2016). Forti et al. (2010) reported no significant differences in stabilometric variables during exposure to Mozart's Jupiter Symphony in healthy adults compared to other music genres; however, vestibular and somatosensory inputs were found to be enhanced. Based on these findings, it was assumed in the present study that the Jupiter composition would not exert a significant direct effect on postural sway. While it has been argued that Mozart music has a positive effect on cognitive perception, especially those involving spatial orientation, studies with various tests have not shown definite effects of classical music (Yoo et al. 2022). Zhu et al. (2008) showed a selective positive effect in event‐related potentials on involuntary attention during listening to the Mozart K.448 Sonata, which was not reflected in behavior. Jaušovec and Habe (2003) found that Mozart's sonata K. 448 affected attention and alertness significantly more than other composers. At the beginning of our study, it was hypothesized that Mozart music presented during the Stroop test would increase attention on the Stroop test and that maintaining voluntary attention on the cognitive task would improve balance. However, no difference in postural sway was observed between the music and control groups.

In addition, there was no effect on Stroop response time when Mozart music was compared with the silent environment. Kim (2022) found that music evoking negative emotions decreased Stroop accuracy, while Mozart music paired with positive emotions increased Stroop response accuracy, supporting the arousal‐mood hypothesis of music in cognitive tasks. In our study, Mozart music had no effect on Stroop performance during the dual task. At the same time, there was no effect on static balance performance during the cognitive task.

In the study by Bruce et al. (2019), in healthy young adults, multi‐talker babble noise presented during a dual task led to a decrease in participants' cognitive task performance. Schlittmeier et al. (2015) showed that Stroop performance was negatively affected during 60 dBA acoustic stimuli consisting of speech noise and traffic noise. In contrast, in the present study, using multi‐talker babble noise at 7 dB SNR in dual task, noise had no effect on Stroop test performance. However, the mean postural sway velocity during the cognitive task in the noisy environment decreased compared to the silent environment. This may be related to the perception of “stress” in noise as opposed to music. Chajut and Algom (2003) suggested that selective attention increases in the presence of an environmental stressor. However, Starcke et al. (2016) showed that stress negatively affected Stroop test performance and executive functions. In addition, multi‐talker noise causes higher cognitive demand than different types of noise (Ishikawa et al. 2023). In our study, noise‐induced stress did not impair cognitive task performance; however, it was associated with a reduction in postural sway. This finding may be explained by the Constrained Action Hypothesis, which suggests that under increased cognitive load, balance control shifts to more automatic processes. Both the cognitive task and the multi‐talker background noise in the participants' native language increased the cognitive demand, and as a result, postural control was transferred to automatic processes. Notably, this effect was observed only in the noisy environment. There was no difference between the effects of the dual task on postural sway in the noisy and musical environments. Our findings revealed that noise stimulus positively affected static balance performance by decreasing postural sway during the cognitive task.

Interestingly, unlike much of the existing literature (Lacour et al. 2008; Lajoie et al. 2016; Waer et al. 2023), dual‐task conditions in the present study resulted in increased postural sway in healthy adults, both on firm and foam surfaces. This finding suggests that the cognitive task may impair motor performance due to cognitive‐motor interaction. Cognitive effort during the inhibition control task explains the impairment of postural sway (increased sway velocity) according to the nonlinear interaction model (Lacour et al. 2008). While some studies in the literature support the constrained action hypothesis (Lajoie et al. 2016), others have shown that simultaneous cognitive‐motor tasks can disrupt postural control due to competition for limited cognitive resources (Ceyte et al. 2014). The difference between the findings was explained by the difference in the attention manipulation levels of the tasks given in the studies. In cognitive motor dual‐task studies, the priority of young individuals is cognitive task performance. The fact that the cognitive task increased postural sway in our study suggests cognitive task primacy in the relatively easy balance conditions of healthy young adults. Although in young adults cognitive and attentional capacities can be shared smoothly in easy motor tasks, conscious balance maintenance led to increased postural sway when the postural task became difficult, for example when proprioceptive information was unstable. In this study, although balance performance did not reach a level indicating a risk of falling or abnormal postural sway, an increase in sway was observed during the dual‐task condition on the foam surface. This increase was greater than that observed on the firm surface, which provides a more stable postural context. The absence of reliable proprioceptive input from the compliant surface likely led individuals to engage more conscious control strategies to maintain balance. Consistent with our findings, previous studies have reported that postural stability tends to decline under more challenging postural conditions when a cognitive task is performed simultaneously (Lacour et al. 2008). Moreover, more demanding motor tasks, such as walking, have been shown to prompt healthy young adults to prioritize balance maintenance (Small and Neptune 2022).

### 4.1 Limitations

One of the limitations of this study is that the time interval over which swaying was assessed was limited to three recordings of 10 s each. Different outcomes may result from recordings that are either longer or brief. In addition, the inclusion of more diverse acoustic stimuli or the use of different cognitive tasks that engage different functions of the cognitive system may alter the results. These are considered other limitations of this study. However, it should be emphasized that it cannot be known whether the fact that the assessment was performed only in a static position would be reflected in gait or other dynamic motor tasks.

## 5 Conclusions

In this study, neither attentional performance nor postural sway was affected by the dual task performed with relaxing music. Postural sway decreased during the inhibition control task in a noisy environment, which was seen as an external stressor, but participants' cognitive performance did not change. The increased cognitive demand for selective attention required for inhibition control in the presence of multi‐talker noise resulted in the shift of postural control to automatic processes in both postural conditions (firm surface and foam surface). These findings suggest that different acoustic stimuli have different effects on the balance system in the multitasking conditions similar to those encountered in daily life. In future studies, it is recommended to examine the relationships between auditory processing skills and balance skills and to investigate their effects on rehabilitation processes. It is recommended that future studies evaluate the effects of a non‐fatiguing duration in multitasking on different sample populations, the effects of different posturographic parameters, and expanding task and acoustic background stimulus diversity.

## Author Contributions

**Selin Sarıçamlık**: investigation, data curation, methodology, writing – original draft, writing – review and editing, conceptualization. **Nizamettin Burak Avcı**: formal analysis, methodology, writing – review and editing, data curation. **Öznur Yiğit**: project administration, writing – review and editing, resources, supervision, methodology.

## Ethics Statement

This cross‐sectional study was conducted at Hacettepe University, Faculty of Health Sciences, Department of Audiology, with the approval of the Hacettepe University Non‐Interventional Clinical Research Ethics Committee (GO 22/974) in accordance with the Principles of the Declaration of Helsinki. All participants signed an informed consent form that included the purpose of the study, its significance, and answers to possible questions.

## Conflicts of Interest

The authors declare no conflicts of interest.

## Peer Review

The peer review history for this article is available at https://publons.com/publon/10.1002/brb3.70750.

## Data Availability

The original data supporting the findings of this study are available upon request from the corresponding author. The data are not publicly available due to privacy or ethical restrictions.
